# Functional Assessment of EnvZ/OmpR Two-Component System in *Shewanella oneidensis*

**DOI:** 10.1371/journal.pone.0023701

**Published:** 2011-08-23

**Authors:** Jie Yuan, Buyun Wei, Miaomiao Shi, Haichun Gao

**Affiliations:** Institute of Microbiology, College of Life Sciences, Zhejiang University, Hangzhou, Zhejiang, China; University of Birmingham, United Kingdom

## Abstract

EnvZ and OmpR constitute the bacterial two-component signal transduction system known to mediate osmotic stress response in a number of Gram-negative bacteria. In an effort to understand the mechanism through which *Shewanella oneidensis* senses and responds to environmental osmolarity changes, structure of the *ompR-envZ* operon was determined with Northern blotting assay and roles of the EnvZ/OmpR two-component system in response to various stresses were investigated with mutational analysis, quantitative reverse transcriptase PCR (qRT-PCR), and phenotype microarrays. Results from the mutational analysis and qRT-PCR suggested that the EnvZ/OmpR system contributed to osmotic stress response of *S. oneidensis* and very likely engaged a similar strategy employed by *E. coli*, which involved reciprocal regulation of two major porin coding genes. Additionally, the *ompR-envZ* system was also found related to cell motility. We further showed that the *ompR-envZ* dependent regulation of porin genes and motility resided almost completely on *ompR* and only partially on *envZ*, indicating additional mechanisms for OmpR phosphorylation. In contrast to *E. coli* lacking *ompR-envZ*, however, growth of *S. oneidensis* did not show a significant dependence on *ompR-envZ* even under osmotic stress. Further analysis with phenotype microarrays revealed that the *S. oneidensis* strains lacking a complete *ompR-envZ* system displayed hypersensitivities to a number of agents, especially in alkaline environment. Taken together, our results suggest that the function of the *ompR-envZ* system in *S. oneidensis*, although still connected with osmoregulation, has diverged considerably from that of *E. coli*. Additional mechanism must exist to support growth of *S. oneidensis* under osmotic stress.

## Introduction

Osmotic stress caused by changes of environmental osmotic strength is among the environmental stresses of great physiological relevance to microbes [1]. To cope with osmotic stress, bacteria have developed a number of strategies for effective adaptation. These strategies and their underlying mechanisms have been extensively reviewed [2]–[3]. In *Escherichia coli*, it is known that the EnvZ/OmpR two-component system plays a central role in mediating signal transduction in response to osmotic stress [4]–[6]. EnvZ is a transmembrane histidine kinase that monitors environmental osmolarity. At high osmolarity, EnvZ autophosphorylates and transfers the phosphoryl group to the response regulator OmpR, leading to formation of phosphorylated OmpR (OmpR-P). OmpR-P then binds to the promoter regions of outer membrane porin genes *ompF* and *ompC* and differentially modulates their expression according to the cellular OmpR-P level [7]. EnvZ also acts as a phosphatase that dephosphorylates OmpR-P once the osmotic stress fades away. Thus, environmental osmolarity affects the porin composition by the sum of EnvZ kinase and phosphatase activities *in vivo* [8]. In recent years, the regulatory scope of the EnvZ/OmpR system has been extended to genes related to virulence in *Shigella flexneri*, fatty acid receptor, peptide permease, and flagella in *E. coli*, as well as acid shock and stationary-phase acid tolerance response in *Salmonella* [9]–[15]. Deletion of EnvZ/OmpR was reported to have an impact on expression of more than 100 genes in *E. coli*, causing drastic changes in cell growth and important cell functions such as metabolism and motility [16].

*Shewanella oneidensis* MR-1, a facultative anaerobic γ-proteobacterium, is widely distributed in nature [17]. For more than a decade, this bacterium has received intensive studies owing to its remarkably diverse respiratory capacities and the potential for environmental remediation [18]. Despite the grand effort to characterize the transcriptomic responses triggered by various environmental stresses [19]–[22], little is known about how this bacterium senses these insults and modulates gene expression in response. Even though it is believed to posses more than 130 two-component proteins for signal transduction and gene regulation [18], to our knowledge only one pair of the known two-component systems, namely the Arc system have been characterized in *S. oneidensis* to date [23]–[25]. The Arc system in *S. oneidensis* differs substantially from that in *E. coli*, in terms of the structure (no full length ArcB homologue), regulon, and physiological function [23]–[25].

In this study, we chose to investigate the EnvZ/OmpR two-component system in *S. oneidensis* to address whether this system 1) is of general importance to signal perception and transduction post osmolarity changes and beyond, and 2) has deviated structurally and functionally from its counterpart of *E. coli*, like the Arc system did. Our results suggest that, although the *ompR/envZ* null mutant did not show any significant growth defect under conditions tested, these proteins indeed are involved in regulating the expression of porin genes in response to osmolarity changes. Moreover, the EnvZ/OmpR system appears to be important in bacterial tolerance to alkaline environments and affects cell motility. We further showed that the *ompR-envZ* dependent regulation of porin genes and motility resided almost completely on *ompR* and only partially on *envZ*, indicating additional mechanisms for *ompR* phosphorylation.

## Results

### Determination of the structure of the *ompR-envZ* operon

**Figure 1:**
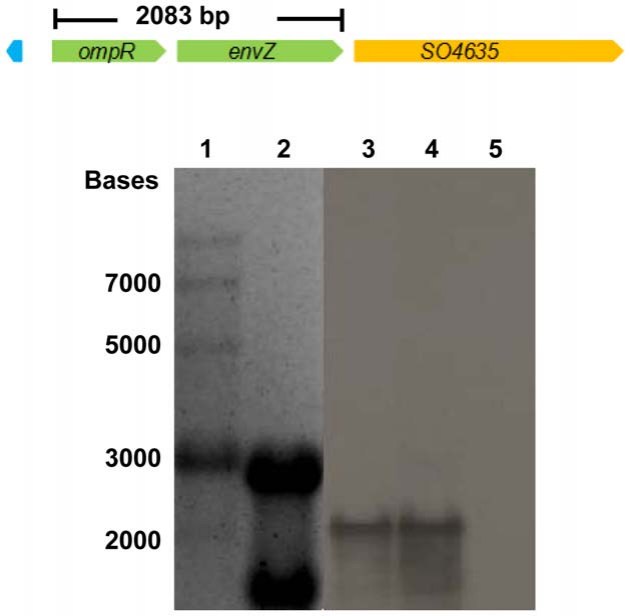
Northern blot assay of the *ompR-envZ* operon. Total RNA used in the analysis was from mid-exponential growing MR-1 cells. Organization of *ompR-envZ* and adjacent genes is shown. Lanes: 1, RNA ladder; 2, total RNA control (same amount of RNA stained with ethidium bromide); 3, RNA hybridized with probe specific for *ompR*; 4, RNA hybridized with probe specific for *envZ*; 5, RNA hybridized with probe specific for *so4635*.

The EnvZ/OmpR system is known to be one of the major regulatory systems controlling the expression of outer membrane porins in response to osmolarity in many Gram-negative bacteria [4], [5]. To determine roles of the EnvZ/OmpR system in *S. oneidensis*, we first determined the structure of the *ompR-envZ* operon. Annotation of the genome sequence for *S. oneidensis* revealed the presence of both *ompR* and *envZ* homologues. Comparison of the deduced amino acid sequence showed that *S. oneidensis* OmpR (242 a. a.) and EnvZ (439 a. a.) share a high degree of identity to their homologues in *E. coli* (79% and 43%, respectively) and *V. cholerae* (241 a. a. 78% and 439 a. a. 42%, respectively), indicating potential for similar biological functions [26]. Although the coding sequences of *ompR* and *envZ* were arranged sequentially as in *E. coli*, appearance of *SO4635*, a methyl-accepting chemotaxis protein coding gene, immediately following *envZ* in the same orientation (Fig. 1) raised the possibility that *ompR*, *envZ* and *SO4635* constitute a distinct type of three-gene operon (as opposed to the usual *ompR-envZ* two-gene operon) in *S. oneidensis*. To determine whether *SO4635* is included in the operon, a Northern blotting assay was performed with RNAs from mid-exponential phase cells. In the analysis, approximately 300 bp internal fragments for each ORF were synthesized by PCR with primers listed in Table S1. As shown in Fig. 1, a∼2.3 kb transcript was detected with probes for either *ompR* or *envZ* whereas no such transcript was found with the probe for *SO4635*. Transcript for *SO4635* alone is about 2 kb long and was not detected at all with the corresponding probe. Therefore, we conclude that *ompR* and *envZ* are co-transcribed as a single operon, while *so4635* is transcribed separately. The function of *SO4635* was not pursuit further.

### Growth of *S. oneidensis* under osmotic stress

**Figure 2:**
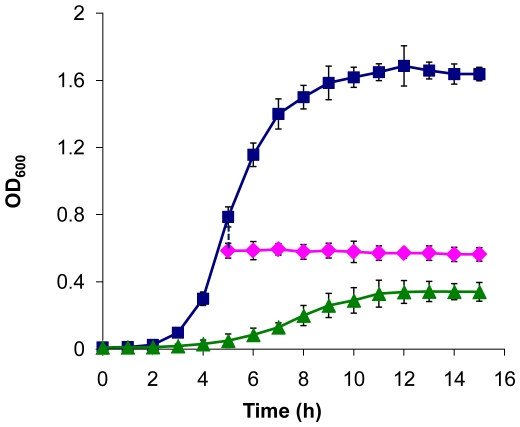
Growth of *S. oneidensis* strains at 30°C in LB medium under normal or osmotic stress conditions. Under normal condition (blue square), osmotic stress condition (20% sucrose) (green triangle), or grown in LB and then transferred to the osmotic shock condition (purple diamond). The growth curves are the average curves for at least three replicate samples and the error bars represent standard error of the mean (SEM).

To probe the effect of osmotic stress on *S. oneidensis* cells, we examined growth of MR-1 in LB medium with 20% of sucrose at 30°C under aerobic conditions. Our results showed that the effect of 20% sucrose on growth of MR-1 was significant (Fig. 2). Even though MR-1 cells were able to survive and grow under osmotic stress, final cell densities are much lower for cultures grown under osmotic stress compared to those under normal conditions, with a maximum cell density of 0.38 (OD_600_). To determine if the final densities represented an irreversible cessation of growth, stressed cells were centrifuged and resuspended in LB (with no sucrose added) to 0.38 of OD_600_. Growth resumed shortly and reached a final densities of 1.8 (optical value of final density under normal conditions) of OD_600_ (data not shown). However, when 20% sucrose (final concentration) was introduced into the cultures of MR-1 at their late exponential phases (OD_600_≈0.8) to impose an osmotic shock, cells stopped growing immediately and the growth never resumed in this sucrose containing medium. Further examination revealed that if the osmotic shock was introduced before the culture reached OD_600_ of 0.38, cell growth can resume in the 20%-sucrose-containing medium, but only to a final OD_600_ of 0.38, indicating that 0.38 of OD_600_ was the maximum cell density permissible by 20% sucrose. Overall, results presented here suggest that the osmotic stress by 20% sucrose prevents *S. oneidensis* cells from growing to high cell densities but such an inhibition is temporary and reversible.

### Construction and characterization of mutants devoid of the EnvZ/OmpR system

To assess the impact of the EnvZ/OmpR system on growth of *S. oneidensis*, the *ompR, envZ,* single and *ompR/envZ* double deletion mutants, designated as HG4633 (Δ*ompR*), HG4634 (Δ*envZ*), and HG4633/4 (Δ*ompR*Δ*envZ*), were created. A mutagenesis system for constructing deletion mutants in *S. oneidensis* MR-1 has previously been developed and successfully utilized [23], [27]. The deletion was confirmed by PCR and DNA sequencing (data not shown).

Physiological role of the EnvZ/OmpR system in *S. oneidensis* was first assessed by growing our mutant strains under either normal or stressed (imposed by 20% sucrose) condition. Strikingly, compared to the parental strain, neither the *S. oneidensis* Δ*ompR*Δ*envZ* double mutant nor either of the two single mutants Δ*ompR* and Δ*envZ* exhibited any noticeable differences in growth, in terms of generation time and maximum cell density (data not shown) under either tested conditions. In contrast, deletion of EnvZ/OmpR was reported to significantly slow down the growth of *E. coli* in LB [16]. These results imply that EnvZ/OmpR system may have diverged considerably between *S. oneidensis* and *E. coli* in terms of their physiological roles.

**Figure 3:**
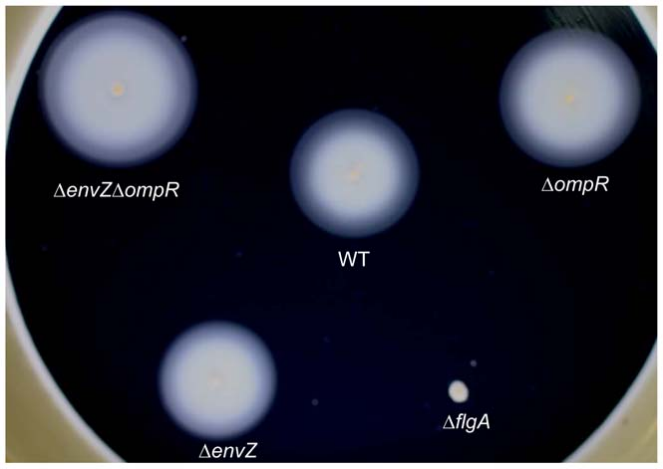
Motility of the Δ*envZ*, Δ*ompR*, Δ*envZ*Δ*ompR* mutants. Both swimming and swarming motility assays were performed. The parental wild type and an aflagellated strain were included as controls. Experiments were performed five times and results were statistically consistent (*p*<0.05). Only swimming results were shown as swimming and swarming are in agreement with each other.

Motility of these mutants was also assayed, as significant down-regulation of the chemotaxis/motility-related genes was previously observed in salt stressed *S. oneidensis* [22], and EnvZ/OmpR was reported to regulate genes involved in flagella synthesis in *E. coli* [16]. Interestingly, our results showed that although motility of Δ*envZ* was the same as its parental strain, Δ*ompR* and the double mutant displayed increased motility (Fig. 3). To ensure that the observed phenotype was due to mutation *per se*, we cloned the operon of *envZ*-*ompR* along with its promoter into pBBR1MCS-2 and introduced the resultant construct into either the *ompR* or the double mutation strain. Swimming motility of the plasmid-borne *ompR* reduced to the level indistinguishable from their parental strains (data not shown). These results suggest that the OmpR may play a role in regulating motility-related genes.

### The *ompR-envZ* operon is induced under osmotic stress

**Figure 4:**
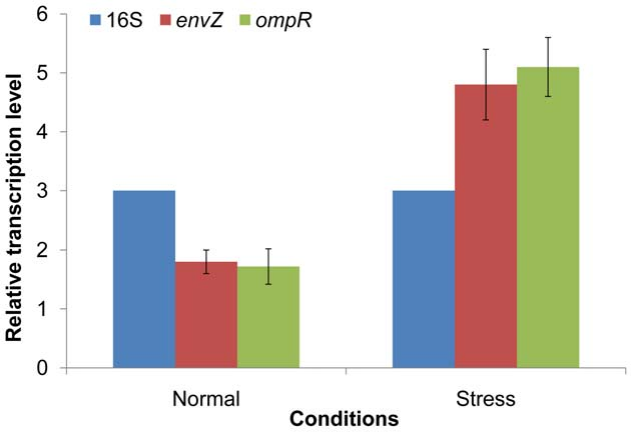
Transcription of *envZ* and *ompR* in *S. oneidensis* under normal and stressed conditions. The relative transcription levels of *envZ* and *ompR* were presented as fold increases with respect to expression of 16S rRNA gene. The results were means plus SEM from three independent experiments.

Apparently, both *ompR* and *envZ* are transcribed in the exponentially growing wild-type cells as their mRNAs allowed determination of the operon structure. However, lack of apparent growth phenotypes of the three *S. oneidensis* mutants under either normal or stressed conditions raised a question about whether the system was osmo-responsive. To test this, we performed qRT-PCR to examine the amount of the *ompR-envZ* transcripts in cells under normal and stressed conditions. Transcripts of *ompR* and *envZ* were virtually the same in wild-type under the same conditions, in agreement with the observation that they are on a single polycistronic mRNA. However, compared to the untreated control, transcription of these two genes was induced approximately 2.8-fold in cells exposed to 20% sucrose (Fig. 4), suggesting potential involvement of *ompR-envZ* in response to osmotic stress.

### *SO0312* and *SO3060* are likely homologues of *E. coli ompC* and *ompF* respectively

The best known osmotic stress response in Gram-negative bacteria is the alteration of membrane porin composition mediated by EnvZ/OmpR system. Hence one of the main goals of this study was to determine the homologues of *E. coli* porin coding genes *ompC* and *ompF* in *S. oneidensis*. Although OmpC and OmpF share substantial similarity in sequence and three-dimensional structure, their expression is regulated reciprocally by the EnvZ/OmpR two-component system upon changes in environmental osmolarity [28]. Elevated osmolarity leads to preferential expression of *ompC* and repression of *ompF*, whereas low osmolarity favors the opposite. *S. oneidensis* has 6 putative outer-membrane porin-coding genes: *SO0312, SO1420, SO1557, SO1821, SO3060,* and *SO3896*. All of them are similar in size (321 – 398 a. a.) and significantly homologous to both *ompC* and *ompF* of *E. coli*.

**Figure 5:**
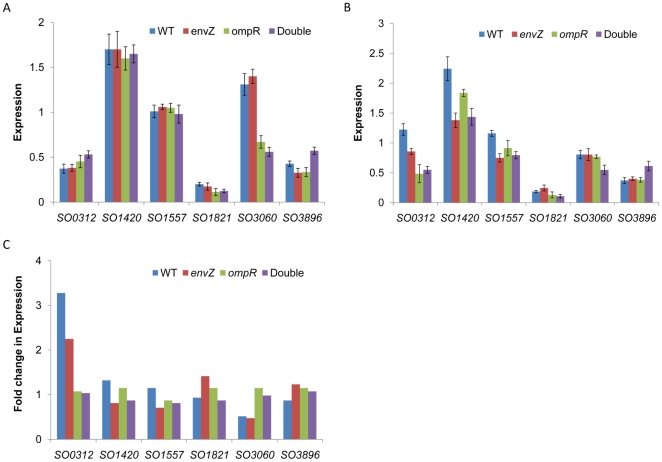
Transcription of six outer membrane porin genes in *S. oneidensis* strains under normal and stressed conditions. All data were normalized to expression of 16S rRNA gene. The absolute expression levels of these genes under normal and stressed conditions were presented in (A) and (B), respectively. The relative transcription levels of these genes were presented as fold changes in each strain between two tested conditions in (C). The results were means plus SEM from three independent experiments.

To determine their involvement in osmotic stress response, expression changes of these genes in wild-type and the three mutant strains (Δ*ompR*, Δ*envZ*, and Δ*ompR*Δ*envZ)* under normal and stressed conditions were compared (Fig. 5). Expression of these genes under normal conditions except for *SO3060* was indistinguishable across all tested strains although their absolute transcript levels varied substantially in each strain (Fig. 5A). The fact that *SO3060* expressed at levels significantly lower in *ompR* and double mutant strains than those in wild-type and Δ*envZ*, suggest that the gene may be under control of OmpR. Under stressed conditions, *SO0312* displayed a responsive expression pattern while the rest 5 genes were not affected (Fig. 5B). We then compared expression of these genes under the two tested conditions as presented in Fig. 5C. Results from the wild-type revealed that transcription of *SO0312* and *SO3060* was drastically affected by the stress imposed by 20% sucrose while the other four genes were hardly responsive. The high osmolarity induced *SO0312* but repressed *SO3060* in wild-type strain, suggesting that *SO0312* and *SO3060* may be the counterparts of *E. coli ompC* and *ompF*, respectively, and *S. oneidensis* very likely employed the same reciprocal transcriptional regulation scheme to modulate membrane porin composition in response to osmotic stress. Strikingly, the same trend was observed in the Δ*envZ* mutant, implicating that the two-component system may still be able to function, at least partially, in the absence of the sensory kinase. In contrast, such response was not found in either Δ*ompR* or the double mutant. These findings indicate existence of EnvZ-independent mechanism for OmpR phosphorylation. Intriguingly, it is know in *E. coli* that ArcB, histidine kinase of the ArcBA two component system can phosphorylate and cross regulate OmpR [29]. To test whether the same mechanism is responsible for OmpR phosphorylation in the absence of EnvZ in MR-1, we examined OmpC expression in mutants devoid of ArcS or both ArcS and EnvZ with qRT-PCR. Additionally, we also employed bacterial two-hybrid system to test interaction of HptA with OmpR. ArcS and HptA are proteins found to function as ArcB in MR-1, with each resembling part of *E. coli* ArcB protein [23]-[25]. Unfortunately, neither was ArcS found to affect OmpC expression in response to osmo-stress, nor was HptA detected to interact with OmpR (data not shown), suggesting that the *S. oneidensis* counterpart to *E. coli* ArcB may not be able to cross-phosphorylate OmpR.

### Functional analysis of the EnvZ/OmpR system

**Figure 6:**
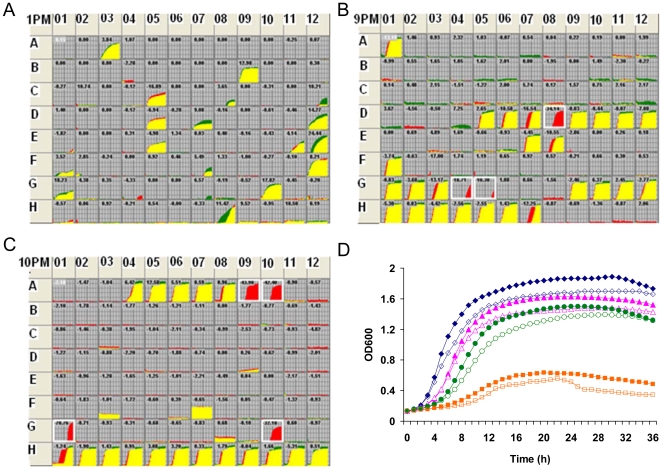
Involvement of the EnvZ-OmpR system in metabolism and stress response. (A, B, C) The *envZ*/*ompR* mutation strain was subjected to PM analysis (3 PMs). The PM kinetic profile shown by comparing the *ompR-envZ* mutant (green) and its wild-type parental strain (red). Red indicates a stronger response by MR-1, and green indicates a stronger response by the mutant; when the two strains have equivalent metabolism or equivalent growth responses in a well, the red and green kinetic graphs overlap and are yellow. A box around a growth curves indicates a significant difference in response. (D). Independent growth studies in LB with various concentrations of sodium sulfate. 2%, diamond; 3%, triangle; 4%, cycle; 5%, square. In all cases, MR-1, closed; *ompR-envZ-*, open. Experiments were performed three times independently and consistent results were obtained. For clarity, error bars (SEM, < 5%) were omitted in the figure.

The results presented above indicated that the EnvZ/OmpR system probably still contributes to osmotic stress response (very likely by modulating membrane porin composition) and affect cell motility. However, under the conditions tested, lack of EnvZ/OmpR did not cause a pleiotropic effect as observed in *E. coli* [16]. To evaluate impact of the EnvZ/OmpR system on metabolism of *S. oneidensis* under a broader set of conditions, capacity of the wild-type, Δ*ompR*, Δ*envZ*, and Δ*ompR*Δ*envZ* strains to metabolize 95 different carbon sources was tested using PM1 phenotype microarrays from Biolog (information about carbon sources at http://www.biolog.com/pdf/PM1-PM10.pdf). All these strains displayed positive reaction with carbon sources A03 (N-acetyl-D-glucosamine), B09 (D, L-Lactic acid), C05 (tween 20), D05 (tween 40), D12 (uridine), E05 (tween 80), E11 (2′-deoxyadenosine), E12 (adenosine), F12 (inosine), G10 (methylpyruvate), and H08 (pyruvic acid) (Fig. 6A). However, no difference was observed between profiles obtained from the wild-type and any of the mutants. In contrast, the same phenotype microarrays revealed that the *E. coli* EnvZ/OmpR mutants displayed increased consumption of D-glucose, D-fructose, mannitol, and N-acetyl-D-glucosamine [30]. Collectively, these results indicate that that the EnvZ/OmpR system is unlikely to be involved in regulation of carbon metabolism in *S. oneidensis*.

We then examined whether the EnvZ/OmpR system has an impact on bacterial response to various stresses using PM9 and PM10 phenotype microarrays. Lack of significant difference in profiles between the three mutants used (data not shown) indicates that both the sensor (EnvZ) and the regulator (OmpR) appear to be essential to the functionality of the two-component system in the context of the PM analysis. To be concise, we used the phenotype microarray results of Δ*ompR*Δ*envZ* strain to represent those of all mutant strains unless otherwise noted. As shown in Fig. 6B and C, the mutant was notably hypersensitive to 5% sodium sulfate (PM09, D08), 200 mM sodium phosphate at pH 7.0 (PM09, G04), 20 mM sodium benzoate at pH 5.2 (PM09, G05), pH 8.5 (PM10, A09), pH 9 (PM10, A10), pH 9.5 + anthranilic acid (PM10, G01), and pH 9.5 + tryptamine (PM10, G10). In contrast to *E. coli ompR-envZ* mutants, the *S. oneidensis ompR-envZ* mutants were not hypersensitive to ethylene glycol. It is worth noting that most of these hypersensitivities were observed in alkaline environment, suggesting that the two-component system may be involved in bacterial response to alkaline stresses. Interestingly, in *Salmonella enteric* the system plays a significant role in the acid stress response [31].

To confirm the phenotypes revealed by PM analysis, hypersensitivity of the mutation strains to 5% sodium sulfate was assessed by an independent growth study. All the wild-type and mutant strains were investigated in LB supplemented with sodium sulfate at concentrations of 2, 3, 4, and 5% (Fig. 6D). Consistent with the PM analysis, 5% sodium sulfate was found to inhibit growth of the bacteria, with a more significant impact on the mutant strain. Interestingly, in the growth study, the mutant displayed hypersensitivity to all tested concentrations of sodium sulfate, although to much lesser extent at lower concentrations compared to 5%. The fact that PM analysis failed to reveal these milder effects could probably be attributed to the limited sensitivity of the colorimetric mechanism employed by the approach. Nonetheless, our results suggest that even though PM analysis may not be suitable to pick up significant subtle differences, it provides a valuable tool to examine over thousands of cellular phenotypes in a high-throughput fashion and provide meaningful multi-dimensional biochemical profiles of organisms. These profiles alone, or combined with transcriptomes and proteomes, will greatly facilitate the future researches in all directions.

## Discussion

Two-component signal transduction systems composed of a sensory histidine kinase and a response regulator are broadly utilized by bacteria for effective acclimation to environmental changes. Given the number of studies aiming to dissect stress responses of *S. oneidensis* performed, it is surprising that the Arc two-component system is the only one being scrutinized [23]–[25]. Additionally, the Arc system is so atypical that it does not only replace the full length ArcB with two proteins but also differs substantially in physiological function from its *E. coli* counterpart. Thus, investigation into other two-component systems of *S. oneidensis* seems demanding.

Among the most extensively studied, EnvZ/OmpR is known to govern the response to osmotic changes by modulating expression of porin coding genes *ompC* and *ompF* and thereby outer membrane permeability [32]. The objective of present work was to investigate whether the EnvZ/OmpR system is conserved structurally and functionally in *S. oneidensis*. The Northern blotting assay revealed that genes coding for EnvZ and OmpR constitutes an *ompR-envZ* operon which was transcribed as a single polycistronic mRNA. Transcription of this operon was found to increase ∼2.8 fold under osmotic stress, consistent with the system being involved in osmotic stress response. In two-component systems, differences at the transcriptional level have been largely overlooked as the phosphorylation states of the response regulator was widely accepted as the determining factor for functionality [33]–[34]. Our results indicated that increased transcription of *ompR*-*envZ* might also be a strategy that *S. oneidensis* cells adopt to tackle the osmotic stress problem. An exemplary strategy of such kind was employed by the two-component system NRI-NRII: members of the NRI regulon are known to require different amount of the response regulator for activation or repression, so different response can be triggered at different expression level of NRI-NRII [35]. By this line of reasoning, the increment observed in *envZ*/*ompR* transcription could also be perceived as hinting an OmpR regulon of a considerable size.

In addition, we presented evidence that, among the 6 putative porin coding genes found in the *S. oneidensis* genome, *SO0312* and *SO3060* were very likely counterparts of *E. coli ompC* and *ompF*, respectively. We showed that expression of these two genes responded reciprocally to osmotic stresses in an OmpR-dependent manner, while only partial dependency was observed for EnvZ. In addition, motility was also found to increase in the absence of OmpR but not EnvZ. These results on one hand suggest that EnvZ/OmpR is still linked to osmotic stress response, and indicate the presence of additional mechanisms responsible for OmpR phosphorylation on the other hand. Phosphorylation of OmpR in the absence of EnvZ can be attributed to cross-regulation among two-component systems [36]. Although ArcB was known to regulate OmpR in *E. coli* [29], such effect was not observed in our study. It is not surprising however, given that no apparent orthologue of ArcB exits in MR-1 and its role is played by two proteins together. Moreover, MR-1 possesses a much larger arsenal of two-component systems compared to *E. coli* [18]; it is poised to employ a more sophisticated cross-talking signal transduction network. In addition to non-cognitive histidine kinases, even non-enzymatic phosphorylation agents such as acetyl phosphate, a global signal that feeds into various two-component systems [37], could also be responsible for OmpR phosphorylation. This may be particularly worth noting because acetyl phosphate was involved with the EnvZ/OmpR system as a phosphoryl group donor and its level correlated with the level of phosphorylated OmpR in *E. coli* [38]–[39]. Although phosphorylation of OmpR by agents other than EnvZ is not expected to be as efficient as EnvZ, dephosphorylation of OmpR is also greatly reduced in Δ*envZ* due to the lack of phosphatase activity of EnvZ. Hence we postulate that such EnvZ-independent phosphorylation could still support OmpR-P accumulation to physiologically relevant levels to regulate genes such as *ompC* and *ompF* in the absence of EnvZ.

In contrast to the significant growth impairment reported for a *E. coli* strain devoid of the entire system [16], none of the three deletion mutants (Δ*ompR*, Δ*envZ*, Δ*ompR*Δ*envZ*) of *S. oneidensis* displayed noticeable growth defect compared to the parental wild type strain even under the osmotic stress (by 20% sucrose). This apparent robustness of growth to loss of EnvZ/OmpR could potentially be due to additional mechanisms coping with osmotic changes. For example, RcsB-RcsC was reported to respond to osmolarity in *Salmonella typhi* [40], and OmpR-independent mechanism influencing *ompC* and *ompF* expression was also suggested to exist in *E. coli* [41]. Given the presence of shewanellae in a large spectrum of environmental conditions that covering a broad osmolarity range [42]–[48], it is not surprising if organisms in this genus have developed sophisticated multi-tiered defense mechanism to deal with osmotic changes. Moreover, as far as the data from Phenotype Microarrays were considered, the Δ*ompR*Δ*envZ* mutants of *E. coli* and *S. oneidensis* exhibited drastic differences [27], suggesting that the function of EnvZ/OmpR has diverged significantly between the two organisms. In particular, the apparent importance of EnvZ/OmpR under alkaline conditions in *S. oneidensis* indicates that this system has shifted focus to a different set of environmental insults and acquired new roles.

In conclusion, we show in this work that the EnvZ/OmpR system in *S. oneidensis*, despite a few similarities, departs significantly from that system in *E. coli*. We speculate that these findings reflect a general trend of functional divergence of two-component systems in *S. oneidensis* compared to the Gram-negative model bacterium *E. coli*. Although two-component systems are ubiquitous signal transduction modules to bacteria, the functions of specific two-component systems however, can be tailored in different organism in a natural-habitat-specific manner.

## Methods

### Bacterial strains, plasmids, and culture conditions

A list of all bacterial strains and plasmids used in this study is given in Table 1. *S. oneidensis* and *E. coli* strains were grown in Luria-Bertani (LB, Difco, Detroit, MI) medium at 30 and 37°C, respectively. When needed, the growth medium was supplemented with antibiotics at the following concentrations: ampicillin at 50 µg/ml, and gentamycin at 15 µg/ml. The suicide vector pDS3.0 has been described elsewhere [27].

**Table 1:** Strains and plasmids used in this study.

Strain or plasmid	Description	Reference or source
*E. coli* strain		
WM3064	Donor strain for conjugation; Δ*dapA*	[49]
*S. oneidensis* strains		
MR-1	Wild-type	Lab stock
HG4633	*ompR* in-frame mutant derived from MR-1; Δ*ompR*	This study
HG4634	*envZ* in-frame mutant derived from MR-1; Δ*envZ*	This study
HG4633-4	*ompR-envZ* in-frame double mutant derived from MR-1; Δ*ompR*Δ*envZ*	This study
HG3256	*flgA* in-frame mutant derived from MR-1; Δ*flgA, aflagelated*	[50]
Plasmids		
pDS3.0	Ap^r^, Gm^r^, derivative from suicide vector pCVD442	[27]
pDS3-OMPR	pDS3.0 containing the PCR fragment for deleting *ompR*	This study
pDS3-ENVZ	pDS3.0 containing the PCR fragment for deleting *envZ*	This study
pDS3-OED	pDS3.0 containing the PCR fragment for deleting *ompR* and *envZ*	This study
pBBR1MCS-2	Broad host Km^r^ vector used for complementation	[51]
pBBR-OED	pBBR1MCS-2 containing the *envZ*-*ompR* operon with its promoter	This study

### Construction of *S. oneidensis* in-frame deletion strains

The mutagenesis method for both single and double in-frame deletion mutations were the same except that different sets of primers were used to generate insertion fragments. Primers used in this study were listed in Table S1 of the supporting information. Below the method used for construction of the *ompR* in-frame deletion mutation strain HG4633 (Δ*ompR*) was briefly described.

Two fragments flanking *ompR* were amplified by PCR with primers SO4633-5-F and SO4633-5-R, primers SO4633-3-F and SO4633-3-R respectively, and joined together by the second round PCR with primers SO4633-5-F and SO4633-3-R. The resulting fragment was digested with *Sac*I (New England Biolabs, Beverly, MA) and then ligated into *Sac*I site of pDS3.0 treated with the shrimp alkaline phosphatase (Roche Diagnostics, Mannheim, Germany), resulting in the plasmid pDS3-OMPR. *E. coli* WM3064 cells containing pDS3-OMPR were used for conjugal transfer of pDS3-OMPR to *S. oneidensis* MR-1 as described previously [27]. In-frame deletion mutations were screened by colony PCR amplification and verified by DNA sequencing of the PCR-amplified DNA fragment containing the mutated region. For strains HG4634 (Δ*envZ*) and HG4633/4 (Δ*ompR*Δ*envZ*), primers used were: SO4634-5-F and SO4634-5-R, SO4634-3-F and SO4634-3-R; SO4633-5-F and SO4633-5-R, SO4634-3-F and SO4634-3-R, respectively.

### Growth of wild-type and mutant strains on normal and osmotic stress conditions

A single colony of each *S. oneidensis* strain was used to inoculate 5 ml of LB in 50 ml plastic tubes and grown overnight at 30°C (optimal growth temperature) on a rotary platform (200 rpm). This culture was then used to inoculate 30 ml of LB medium with or without 20% sucrose pre-warmed to 30°C in a 250 ml shake flask at an OD_600_ of 0.01 and the flask was shaken on a rotary platform (250 rpm) at 30°C. For the osmotic shock, cells were grown to exponential phase and diluted with prewarmed LB supplemented with 50% sucrose to the final sucrose concentration of 20%. For RNA work, cells before and 10 min after addition of sucrose were pelleted by centrifugation for 30 seconds, frozen immediately in liquid nitrogen, and then stored at −80°C. Three individual cultures were grown in parallel as biological replicates. Growth was measured every 30 min for normal condition and every 4 h for stress condition. Three individual cultures for each strain were grown simultaneously. Growth was measured every 30 min and 4 h under normal and stress conditions, respectively.

### Swarming and swimming motility assay and mutation complementation

A fresh colony of tested strains was grown to an OD_600_ of 0.8 in LB media. The cultures (1 µl) were spotted onto a swarming LB plate (0.5% agar) or stabbed into a swimming LB plate (0.2% agar). All plates were incubated at the room temperature for 48 h. For complementation, DNA fragments containing *envZ* and *ompR* as well as their promoter were generated by PCR amplification with MR-1 genomic DNA as the template using primers SO4633/4-COM-F and SO4633/4-COM-R, respectively as listed in Table S1. These fragments were digested with *Sac*I and ligated to *Sac*I-digested pBBR1MCS-2 to form pBBR-OED, which was electroporated into WM3064. Introduction of pBBR-OED into all mutants constructed in this study was done by conjugation, and kanamycin-resistant colonies were selected. The presence of pBBR-OED in the mutants was confirmed by plasmid purification and restriction enzyme digestion.

### Northern blot hybridization

In order to determine the genes that constitute the *ompR* operon, Northern blotting was performed with total RNA extracted from exponentially growing *oneidensis* MR-1 cells (OD_600_≈0.4). RNA was extracted using Trizol (Invitrogen) and RNeasy kit (Qiagen) as described previously [19]. All chemicals used in this experiment were obtained from Roche Diagnostics (Roche Diagnostics, Indianapolis, IN) and every step was performed according to the manufacturer's instructions unless otherwise indicated. Digoxigenin (DIG)-labeled DNA probes were generated using the PCR DIG Probe synthesis kit. The primers used to generate probes of *ca.* 300 bp for *ompR*, *envZ* and *so4635* were listed in Table S1. 15 µg (per lane) of the same total RNA used in RT-PCR was electrophoresed on a 1.2% agarose gel in 1x MOPS containing 2% formaldehyde. Gels were run for 5 h at 55 V in the cold. Lanes corresponding to the molecular weight markers (RNA ladder, New England Biolabs, Beverly, MA) and control RNA sample were cut out, stained with ethidium bromide and photographed under UV light. Transfer of RNA on the rest of the gel to nylon membranes was done by upward capillary blotting with 20x SSC. The RNA was then bound to the nylon membrane using Stratalinker 1800 at 120 mJ for 1 min. After prehybridization, hybridization was performed overnight at 50°C using approximate 4 ng of labeled probe per ml of DIG hybridization buffer. Washing of the membrane and detection of specific transcripts on the blots were carried out using washing and blocking reagents and the DIG luminescent detection kit. Signals were visualized by exposure to X-ray film (Kodak, Rochester, NY). The size of the target mRNA was estimated from the sizes of 16S and 23S rRNA, and the RNA ladder detected by ethidium bromide staining before membrane transfer.

### Quantitative RT-PCR (qRT-PCR)

RNA templates for the analysis were extracted from the wild-type and mutant cells collected 10 minute after 20% sucrose was introduced. Primers were designed using Omiga software (Oxford Molecular Ltd., San Diego, CA) and were synthesized by Applied Biosystems (Table S1). PCR products amplified from these ORFs were single-band fragments of 99–101 bp in length, as confirmed by agarose gel electrophoresis. A 100-bp fragment of the *arcA* gene, which was amplified by PCR with genomic DNA as the template, was used to construct the standard curve. The reaction was performed with 50 cycles of 30 s at 94°C, 30 s at 55°C, and 1 min at 72°C and monitored in an iCycler iQ real-time PCR detection system (Bio-Rad, Hercules, CA). The transcriptional levels of target genes were normalized against the expression of 16S rRNA gene as internal control. The expression of each gene was determined from three replicates on a single qRT-PCR experiment.

### Bacterial two-hybrid assay

The BacterioMatch II Two-Hybrid system was used to investigate protein-protein interaction between HptA and OmpR *in vivo* in *E. coli* cells according to manufacturer's instructions. Briefly, the genes *htpA* and *envZ* (without the first 114 bases encoding the first membrane-spanning region), were cloned into plasmid pBT separately, and *ompR* gene was cloned into plasmid pTRG. After verification by sequencing, the resultant plasmids were co-transformed into BacterioMatch II Validation Reporter Competent Cells on M9 salt agar plates containing 25 mg/ml chloramphenicol and 12.5 mg/ml tetracycline with or without 3-amino-1,2,4-triazole (3-AT). And pBT-LGF2, pTRG-Gal11P, empty pBT and pTRG were used as positive and negative controls. The plates were incubated at 37°C for 24 h and then moved to room temperature in a dark location (to preserve the tetracycline) for an additional 16 h. The strength of interaction typically correlated with the ratio of colonies obtained on the selective plated compared to on the non-selective plates. The positive interactions were confirmed by streaking colonies on plates containing both 3-AT and streptomycin (12.5 mg/ml).

### Phenotype Microarrays

Phenotype microarray (PM) plates (Biolog Inc., Hayward, California) were used to examine the involvement of the EnvZ/OmpR two-component system in carbon metabolism and various stresses according to the manufacturer's instruction. The altered phenotypes of the Δ*ompR*, Δ*envZ*, and Δ*ompR*Δ*envZ* mutants were assessed by comparing to their parental strain MR-1. The mutant strains were recorded as a green trace and MR-1 was recorded as a red trace in OmniLog Incubator-Reader. Color-coded kinetic graphs could then be overlaid by the OmniLog PM bioinformatic software, and differences were visualized and quantified according to the manufacturer's instruction. To confirm the PM analysis results, growth of the wild-type and mutation strains in LB supplemented with selected substances identified by PM analysis was investigated.

## Supporting Information

Table S1 **Primers used in this study.**
(PDF)
