# Supplementary material for: Functional Assessment of EnvZ/OmpR Two-Component System in Shewanella oneidensis
Source: PLoS One. 2011 Aug 23;6(8):e23701. doi: 10.1371/journal.pone.0023701 (PMC3160321; doi:10.1371/journal.pone.0023701)
Supplement: Table S1 — Primers used in this study. (PDF) [file pone.0023701.s001.pdf]

TABLE S1. Primers used in this study

Mutagenesis	
SO4633-5-F	TACTCAGAGCTCCCACCAAAG
SO4633-5-R	CCCATCCACTAAGTTTAAACATGCTCGTAGGCGCATATC
SO4633-3-F	TGTTTAAACTTAGTGGATGGGTTTGTGCCCGACGGTGCC
SO4633-3-R	CCGTAGTAGGGAGCTCGTTCG
SO4634-5-F	GGCTATGTGTTTGAGCTCGAC
SO4634-5-R	CCCATCCACTAAGTTTAAACATTCGATACCTGCTTCGCG
SO4634-3-F	TGTTTAAACTTAGTGGATGGGGCCCAAGTTTGGCTACCG
SO4634-3-R	GGAGGCTGCGAGCTCCAGTT
SO0557-5-O	GGGGACAAGTTTGTACAAAAAAGCAGGCTCGGAAAATAAGGGGAAACG
SO0557-5-I	CCAATACTGCGTGACCTGATATTGCACGCGATAAAACG
SO0557-3-O	GGGGACCACTTTGTACAAGAAAGCTGGGTTTATGGCACCCCTAAAGG
SO0557-3-I	ATCAGGTCACGCAGTATTGGGTGCAACTTCTCAAGCAG
SO0557-LF	CCGATAACTCAATTACCACC
SO0557-LR	GCAGGCTCTATGACATCC
SO0557-SF	CGGAAAATAAGGGGAAACG
SO0557-SR	ACAATGCTAAAACCCAATGG
Complementation	
SO4633/4-COM-F	CTCGAGCTCTGTCGCATTCGACTGCCG
SO4633/4-COM-R	AATGAGCTCCCCAATGTCAGCGTTGTCAT
Northern	
SO4633-N-F	TATGCGCCTACGAGCACTCC
SO4633-N-R	AATCCGCGCCAATAGTTCC
SO4634-N-F	GAACACTTAACCATAGTCG
SO4634-N-R	GATCACCATTAAGTACAAGG
SO4635-N-F	CAACCCAAGACAATTACTCG
SO4635-N-R	AGTTCATCCGCTTCTTGC
qRT-PCR	
16S-RT-F	CAGCACAAGTGAGTTTACTC
16S-RT-R	GCAGTCGTTTCCAACTGTTA
SO4633-RT-F	GTGATGCTGACGGCTAAGGG
SO4633-RT-R	ATAGTTCCCGCGGGTTAAAC
SO4634-RT-F	GCCGAAGTCCGTGTCACCCA
SO4634-RT-R	CGTTTTGACCAATCAGCGGT
SO3988-RT-F	GTGAATAGCGCTGGTAACGA
SO3988-RT-R	GGCTTACGAGAGAACGGCTG
SO0312-RT-F	AGCTTTGAACGCAGCGAGAG
SO0312-RT-R	CACCTTGCATCACTAAACTG
SO1420-RT-F	GGTGTTAAGCACAGCACTTA
SO1420-RT-R	AGGAGTAATTGTCCATTGCA
SO1557-RT-F	ACGGGTGCTGCATCGAGCGA
SO1557-RT-R	ATAACGTGTCGTTACGACCC

SO1821-RT-F	ATCGGTAAACAATGGGGAGC
SO1821-RT-R	CTTTGTTAAAGGTGTAAACA
SO3060-RT-F	ACTACTGACGTGCTCAATGC
SO3060-RT-R	GCAGCGCCGCGTCAGCACGA
SO3896-RT-F	GGTTCTGGTGATGTGTTTAA
SO3896-RT-R	CTTGTTTAAATACAGTGTCA
Two-hybrid assay	
SO1327-TH-F	GGAATTCTATGGCCACAGTTGAATTAG
SO1327-TH-R	CCGCTCGAGTTAGGCTTTAGCCTGTAAG
SO4633-TH-F	GGAATTCAGATGGGACAAGAAACCTCG
SO4633-TH-R	GACTAGTTCATCGCCGGGCGGCAC
SO4634-TH-F	GGAATTCGGTGTATGTGTTAAAGCCCAG
SO4634-TH-R	GAAGATCTTATTCTCGCGGTAGCCAAAC
